# Supplementary material for: Distinct miRNA Signatures and Networks Discern Fetal from Adult Erythroid Differentiation and Primary from Immortalized Erythroid Cells
Source: Int J Mol Sci. 2021 Mar 31;22(7):3626. doi: 10.3390/ijms22073626 (PMC8037168; doi:10.3390/ijms22073626)
Supplement: Supplementary file 1 [file ijms-22-03626-s001.zip › Supplementary_Tables_20210330.zip!/Table_S01.pdf]

**Table S1. Summary of detected sncRNAs in samples.**
miRNA:microRNA, piRNA: piwi interacting RNA

| Sample name | Known miRNA count | Novel miRNA count | Known piRNA count | Novel piRNA count |
|---|---|---|---|---|
| CD34_E_A | 1101 | 440 | 280 | 1202 |
| CD34_E_B | 1167 | 459 | 228 | 1459 |
| CD34_E_C | 1160 | 400 | 226 | 959 |
| CD34_L_A | 939 | 211 | 336 | 1801 |
| CD34_L_B | 993 | 373 | 145 | 1302 |
| CD34_L_C | 1128 | 292 | 237 | 1047 |
| HUDEP1_E_A | 1124 | 488 | 149 | 1057 |
| HUDEP1_E_B | 1198 | 454 | 152 | 1711 |
| HUDEP1_E_C | 1149 | 692 | 157 | 1816 |
| HUDEP1_L_A | 1013 | 169 | 198 | 835 |
| HUDEP1_L_B | 988 | 299 | 216 | 1653 |
| HUDEP1_L_C | 947 | 447 | 164 | 1734 |
| HUDEP2_E_A | 1097 | 551 | 279 | 947 |
| HUDEP2_E_B | 1065 | 696 | 204 | 1375 |
| HUDEP2_E_C | 1121 | 431 | 224 | 1335 |
| HUDEP2_L_A | 625 | 177 | 214 | 829 |
| HUDEP2_L_B | 967 | 315 | 182 | 976 |
| HUDEP2_L_C | 943 | 260 | 199 | 1011 |
