# Supplementary material for: Distinct miRNA Signatures and Networks Discern Fetal from Adult Erythroid Differentiation and Primary from Immortalized Erythroid Cells
Source: Int J Mol Sci. 2021 Mar 31;22(7):3626. doi: 10.3390/ijms22073626 (PMC8037168; doi:10.3390/ijms22073626)
Supplement: Supplementary file 1 [file ijms-22-03626-s001.zip › Supplementary_Tables_20210330.zip!/Table_S04.pdf]

Table S4. Selection of exemplary miRNAs with relevant previous characterization.

miRNA ID	Comment	Panel*	Reference
miR-451a	The most abundant miRNA in erythrocytes, expression levels increase remarkably during erythroid differentiation, regulated by GATA 1	a, b, c	[17–19]
miR-15b-5p	Upregulated during erythroid differentiation	a, c	[18,41]
miR-16-5p	Upregulated during erythroid differentiation	a, c	[18,41,51]
miR-96-5p	Upregulated during erythroid differentiation, upregulated in adult vs fetal erythroblasts, targets γ-globin, knock-down increases γ-globin expression by 20%	a, b, c, f	[41,28]
miR-22-3p	Upregulated during erythroid differentiation	a, c	[18,41]
miR-182-5p	Abundant in erythroid cells, upregulated in adult vs fetal erythroblasts	a, c, f	[52,66]
miR-4732-3p	Abundant in erythroid cells, regulates the TGF-β signaling cascade	a, c	[52]
miR-223-5p	Downregulated during erythroid differentiation, targets LMO2	a, c	[20]
miR-221-3p	Downregulated during erythroid differentiation, targets c-KIT	a, d, e	[16]
miR-222-3p	Downregulated during erythroid differentiation, targets c-KIT	a, d, e	[16]
miR181-3p	Downregulated during late-stage erythropoiesis, targets Xpo7, regulates enucleation	a, b, c, d, e	[53]
miR-106a	OncomIR, dysregulated by HPV16-E6/E7	d, e	[58,59]
miR-363	OncomIR, dysregulated by HPV16-E6/E7	d, e, f	[58,59]
miR-18b-5p	OncomIR, dysregulated by HPV16-E6/E7	d, e	[58,59]
miR-20b-5p	OncomIR, dysregulated by HPV16-E6/E7	d, e	[58,59]
miR-92a-2-5p	OncomIR, dysregulated by HPV16-E6/E7	d, e	[58,59]
miR-125b-5p	OncomiR	d	[62]
miR-196a-5p	OncomiR	d	[61]
miR-4707-3p	Dysregulated by HPV16-E6/E7	d, e	[64]
miR-143-3p	Tumor supressor	d, e	[61]
let-7 family	Upregulated in adult vs fetal erythroblasts, targets HMGA2	f	[28,66,67]
miR-146a	Upregulated in adult vs fetal erythroblasts, targets γ-globin	f	[28]
miR-150	Upregulated in adult vs fetal erythroblasts	f	[28]
miR-98-5p	Upregulated in adult vs fetal erythroblasts	f	[66]
miR-183-5p	Upregulated in adult vs fetal erythroblasts	f	[66]
miR-23a-5p	Targets KLF3 and SP1, overexpression elevates expression of γ-globin	f	[68]
miR-27a-5p	Targets KLF3 and SP1, overexpression elevates expression of γ-globin	f	[68]
miR-326	Targets KLF1 expression, overexpression elevates expression of γ-globin	f	[69]
mIR-34a	Targets STAT3 and c-KIT, overexpression elevates expression of γ-globin	d, f	[70]
miR-32-5p	Hydroxyurea-induced expression in sickle cell disease patients, targets BCL11A	f	[71]
miR-340-5p	Hydroxyurea-induced expression in sickle cell disease patients, targets BCL11A	f	[71]

* *Panel in Supplementary Figure 3 where the miRNA is plotted in the context of other differentially expressed miRNAs*
